# 

**Published:** 1911-04-15

**Authors:** 


CONTENTS

Event and Comment, - - - - - - - - - - - - - - - 161

Ante-Natal Pathology, - - - - - - - *By N. S. Hoff, D.D.S.* 163

The Advantages of Nitrous Oxid-Oxygen Anesthesia, Especially in Connection with Ultimate Recovery, *By Raymond C. Coburn, M.A., M.D.* 173

Asepsis in Dentistry, - - *By Jos. S. Graham, M.B., M.R.C.S.* 179

The Country Dentist, - - - - - - - - *By W. E. Green.* 191

The Gingival Third, - - - - - - - - *By J. V. Conzett.* 195

Indents - - - - - - - - - - - - - - - - - - - - 205

Announcements - - - - - - - - - - - - - - - - - - 208

